# Overlapping of Independent SARS-CoV-2 Nosocomial Transmissions in a Complex Outbreak

**DOI:** 10.1128/mSphere.00389-21

**Published:** 2021-08-04

**Authors:** Laura Pérez-Lago, Helena Martínez-Lozano, Jose Antonio Pajares-Díaz, Arantxa Díaz-Gómez, Marina Machado, Pedro J. Sola-Campoy, Marta Herranz, Maricela Valerio, María Olmedo, Julia Suárez-González, Víctor Quesada-Cubo, Maria del Mar Gómez-Ruiz, Nieves López-Fresneña, Ignacio Sánchez-Arcilla, Iñaki Comas, Fernando González-Candelas, Sonia García de San José, Rafael Bañares, Pilar Catalán, Patricia Muñoz, Darío García de Viedma,

**Affiliations:** a Servicio de Microbiología Clínica y Enfermedades Infecciosas, Gregorio Marañón General University Hospital, Madrid, Spain; b Instituto de Investigación Sanitaria Gregorio Marañón (IiSGM), Madrid, Spain; c Servicio de Digestivo, Gregorio Marañón General University Hospital, Madrid, Spain; d CIBER Enfermedades Respiratorias (CIBERES), Madrid, Spain; e Servicio de Medicina Preventiva y Gestión de Calidad, Gregorio Marañón General University Hospital, Madrid, Spain; f Servicio Salud Laboral, Gregorio Marañón General University Hospital, Madrid, Spain; g Genomics Unit, Gregorio Marañón General University Hospital, Madrid, Spain; h Instituto de Biomedicina de Valencia-CSIC, Valencia, Spain; i Joint Research Unit, Infection and Public Health, FISABIO-University of Valencia, Institute for Integrative Systems Biology (I2SysBio), Valencia, Spain; j CIBER Salud Pública (CIBERESP), Madrid, Spain; k Gerencia, Gregorio Marañón General University Hospital, Madrid, Spain; l Departamento de Medicina, Universidad Complutense, Madrid, Spain; m CIBER Enfermedades hepáticas y digestivas (CIBERehd), Madrid, Spain; University of Michigan-Ann Arbor

**Keywords:** COVID-19, SARS-CoV-2, nosocomial transmission, genomic epidemiology

## Abstract

SARS-CoV-2 nosocomial outbreaks in the first COVID-19 wave were likely associated with a shortage of personal protective equipment and scarce indications on control measures. Having covered these limitations, updates on current SARS-CoV-2 nosocomial outbreaks are required. We carried out an in-depth analysis of a 27-day nosocomial outbreak in a gastroenterology ward in our hospital, potentially involving 15 patients and 3 health care workers. Patients had stayed in one of three neighboring rooms in the ward. The severity of the infections in six of the cases and a high fatality rate made the clinicians suspect the possible involvement of a single virulent strain persisting in those rooms. Whole-genome sequencing (WGS) of the strains from 12 patients and 1 health care worker revealed an unexpected complexity. Five different SARS-CoV-2 strains were identified, two infecting a single patient each, ruling out their relationship with the outbreak; the remaining three strains were involved in three independent, overlapping, limited transmission clusters with three, three, and five cases. Whole-genome sequencing was key to understand the complexity of this outbreak.

**IMPORTANCE** We report a complex epidemiological scenario of a nosocomial COVID-19 outbreak in the second wave, based on WGS analysis. Initially, standard epidemiological findings led to the assumption of a homogeneous outbreak caused by a single SARS-CoV-2 strain. The discriminatory power of WGS offered a strikingly different perspective consisting of five introductions of different strains, with only half of them causing secondary cases in three independent overlapping clusters. Our study exemplifies how complex the SARS-CoV-2 transmission in the nosocomial setting during the second COVID-19 wave occurred and leads to extending the analysis of outbreaks beyond the initial epidemiological assumptions.

## INTRODUCTION

Whole-genome sequencing allows assessing the diversity acquired worldwide by SARS-CoV-2 and detection of the emergence of variants that spread more successfully (1, 2; https://virological.org/t/preliminary-genomic-characterisation-of-an-emergent-sars-cov-2-lineage-in-the-uk-defined-by-a-novel-set-of-spike-mutations/563). Similarly, genomic analysis has been applied to evaluate local SARS-CoV-2 spread and better understand its transmission dynamics during an outbreak (3, 4; https://www.krisp.org.za/news.php?id=421). Outbreaks in nosocomial settings are of particular relevance, as they may affect vulnerable individuals and health care workers, who themselves may become transmission vectors and are associated with a higher risk of mortality (4–6). In the first pandemic wave, the shortage of personal protective equipment (PPE) coincided with limited guidance on the appropriate control measures regarding nosocomial transmission by SARS-CoV-2, which may explain that most nosocomial outbreaks refer to that first period. In the second wave, the shortage of PPE has been covered and control measures implemented, but diversity of the SARS-CoV-2 circulating strains has increased. Thus, research focused on nosocomial outbreaks remains a priority. The aim of the study was to perform an in-depth analysis of a suspected second-wave nosocomial outbreak using WGS.

## RESULTS

### Description of the outbreak.

Fifteen patients (Table 1) admitted to the gastroenterology ward (non-COVID-19 area) within a 27-day period (15 September to 12 October 2020) were diagnosed with COVID-19, confirmed by positive SARS-CoV-2 reverse transcriptase PCR (RT-PCR). Most of the patients were male. Hypertension, diabetes, and dyslipidemia were the most common comorbidities. Six out of the 15 patients (40%) developed bilateral pneumonia. Lymphopenia (950 mm^3^; IQR, 400 to 1,300 mm^3^) was observed, associated with an elevated inflammatory marker. Forty percent of the patients (6/15) received systemic corticosteroids, and 46.7% required oxygen support. Two patients were admitted to the intensive care unit (ICU) and required invasive mechanical ventilation. COVID-19-related mortality was 26.7% (Table 1). Additionally, positive RT-PCR results were obtained for three health care workers (HCWs) within the same period. A 50-year-old male nursing assistant (morning/night rotating shift), with a medical history of seasonal asthma for which he used inhaled short-acting beta-2 agonist as needed, with excellent control and no clinical exacerbations, and two female nurses aged 36 and 40 with no relevant medical-surgical history. They developed mild symptoms for few days, with positive RT-PCRs on 24 September and 11 October and an antigen test on 12 October, respectively, confirmed by PCR later.

**TABLE 1:** Demographics, clinical characteristics, and outcomes of patients at diagnosis of SARS-CoV-2^*a*^

Patient characteristics (*n* = 15)	Value
Age (median [IQR] [yrs])	67 (51–79)
No. of males (%)	12 (80)
Comorbidities (no. [%])	
Hypertension	10 (66.7)
Diabetes	7 (46.7)
Dyslipidemia	8 (53.3)
Respiratory disease	5 (33.3)
COPD	3 (20)
Obstructive sleep apnea	1 (6.7)
Interstitial lung disease	2 (13.3)
Lung cancer	1 (6.7)
Ischemic heart disease	1 (6.7)
Cancer	6 (40)
Chronic liver disease	4 (26.7)
HIV	2 (13.3)
Myelodysplastic syndrome	1 (6.7)
Radiologic findings (no. [%])	
Pneumonia	6 (40)
Bilateral opacities	6 (40)
Laboratory findings (median [IQR])	
Lymphocyte count (mm^3^)	950 (400–1,300)
Platelet count (mm^3^)	182,500 (116,250–316,250)
Ferritin (mg/dl)	2,032 (466–2,647)
D-dimer (mg/dl)	553 (282–1,642)
CRP (mg/dl_)	4.15 (1.9–9.3)
Treatment	
Systemic corticosteroids (no. [%])	6 (40)
Remdesivir (no. [%])	3 (20)
Tocilizumab (no. [%])	1 (6.7)
Oxygen therapy (no. [%])	7 (46.7)
Nasal cannula (no. [%])	7 (46.7)
High-flow nasal cannula (no. [%])	3 (6.7)
Invasive mechanical ventilation (no. [%])	2 (13.3)
No. of days on invasive ventilation (median [IQR])	7.5 (3)
Clinical outcomes (no. [%])	
ICU admissions	2 (13.3)
Discharge from hospital	10 (66.7)
Death	5 (33.3)
Death related to COVID-19	4 (26.7)

a IQR, interquartile range; COPD, chronic obstructive pulmonary disease; HIV, human immunodeficiency virus; CRP, C-reactive protein; ICU, intensive care unit.

**FIG 1:**
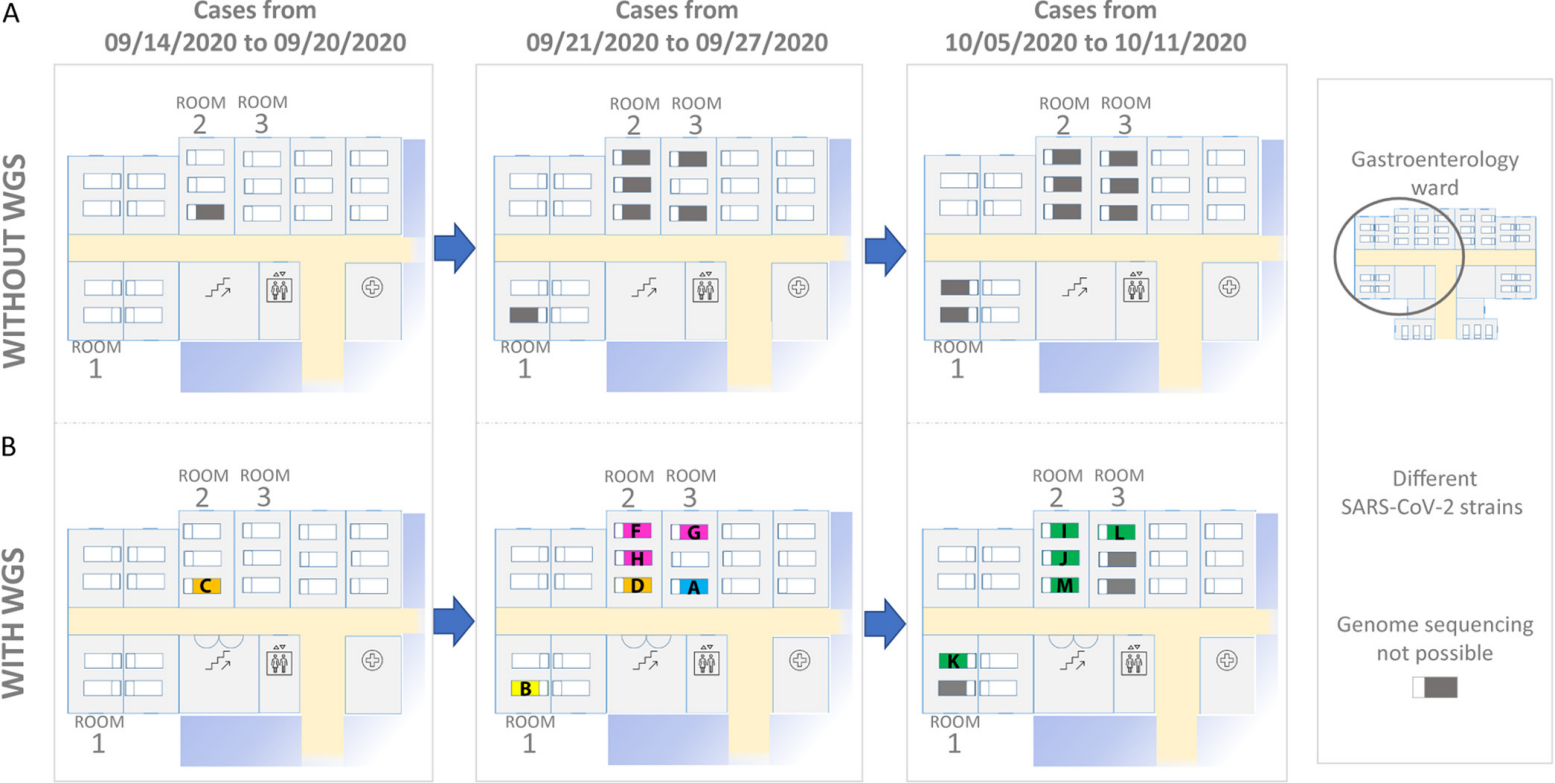
Ward room and bed layout (rooms 1, 2, and 3 in which cases accumulated are numbered). (A) In black, the distribution of SARS-CoV-2 cases throughout three periods (white beds correspond to noninfected cases); they are distributed in three time periods according to the dates for their SARS-CoV-2 diagnostic RT-PCR-positive results. (B) In orange, yellow, pink, blue, green, and black, the distribution of cases after having obtained the genomic data to differentiate between the involved strains (different cases were considered to be infected by the same SARS-CoV-2 strain when the corresponding sequences showed 0 to 1 SNPs between them). The letter codes correspond to the different strains as determined by WGS (Fig. 2).

The gastroenterology ward consists of 12 rooms with 30 beds (Fig. 1A), increased to 37 beds at the beginning of the second wave. All newly diagnosed COVID-19 cases occupied, at different moments, 1 of 3 (two 3 bedrooms and a 2 bedroom; Fig. 1A) of the 12 rooms in the ward.

One hundred and twenty-seven patients were admitted to the non-COVID-19 gastroenterology ward during the study period, from which 38 were at risk. Patients who, at any time during their stay in the hospital, shared a room with a SARS-CoV-2-positive patient were considered at risk {median number (interquartile range [IQR]), 4 days [2.75 to 8.25]} of being infected. Imaging tests results and/or symptoms compatible with COVID-19 allowed making the diagnosis in 14 patients and three HCWs. The remaining case was diagnosed after screening was done due to close contact with a COVID-19 case. Following the protocol established by the hospital, a negative RT-PCR was required to be admitted to a non-COVID ward. Therefore, patients were considered confirmed or probable nosocomial infections; in five cases, the period between their last negative SARS-CoV-2 RT-PCR and a COVID diagnosis was >14 days and 3 to 13 days for the remaining 11 cases.

### Whole-genome sequencing analysis.

**FIG 2:**
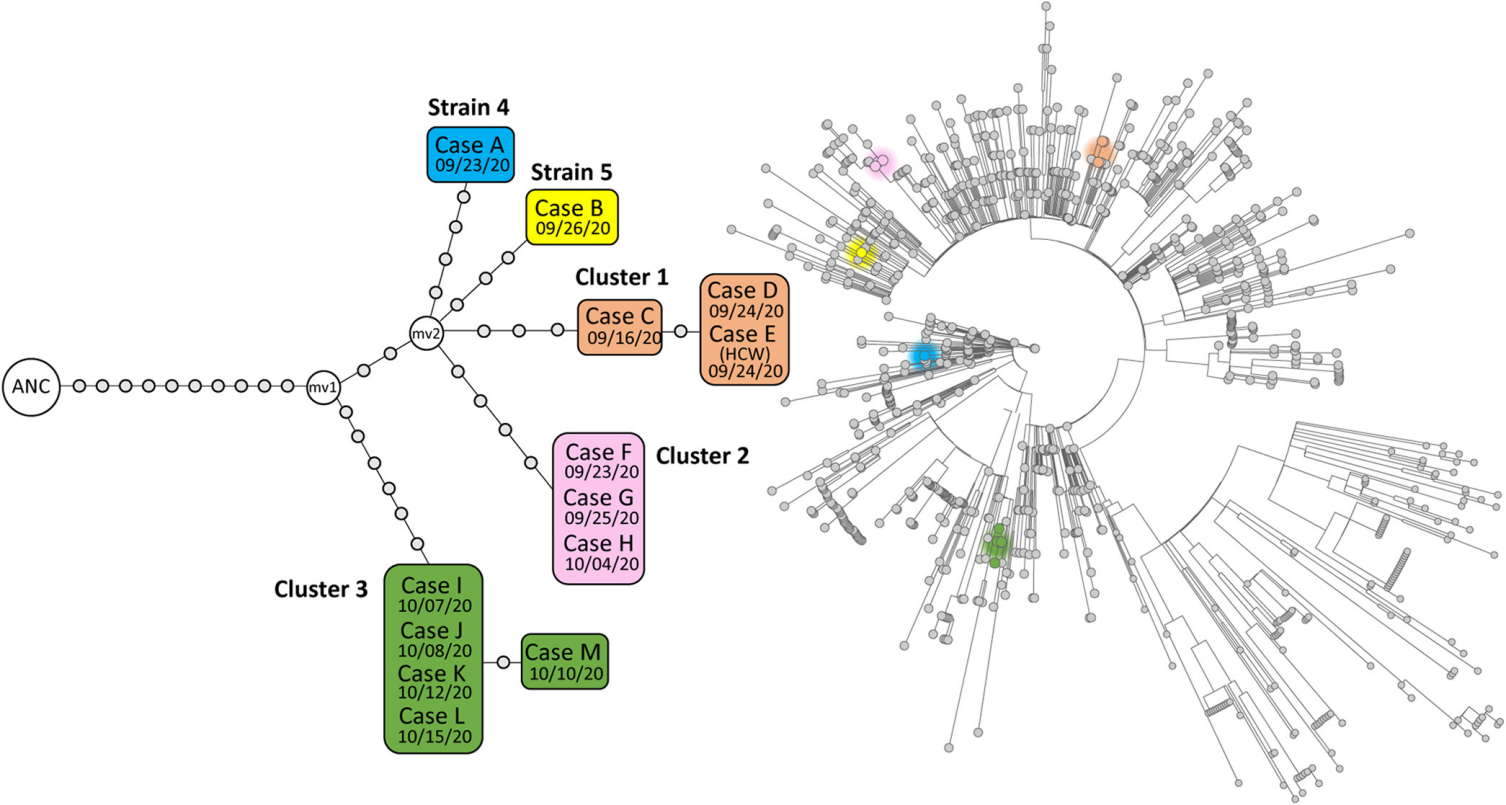
(Left) Network of relationships obtained from whole-genome sequencing analysis for the outbreak strains. Each dot corresponds to a single nucleotide polymorphism. When two or more cases share an identical genome (zero single nucleotide polymorphisms between them), they are included in the same box (each strain represented within a box with a different color). mv, median vector, not sampled recent common ancestor for the two branches; ANC, Wuhan-1 reference strain. (Right) Rooted phylogenetic tree integrating the sequences from the strains in our study (those in the left panel) with the 962 sequences for the same period (1 September to October 15) deposited in https://gisaid.org corresponding to cases from Spain (including 323 from Madrid). Colors in the tree correspond to the colors used in the left panel to differentiate each of the study strains.

Genomic analysis of 13 cases (12 patients and 1 HCW) for which sequencing material was available (mean coverage, 1,502× [range, 686× to 2,247×]; >97% of the positions were covered by >100×) allowed identification of five different SARS-CoV-2 strains, an unexpected diversity (Fig. 2, left). When we integrated the sequences from the five strains identified in the ward, together with 962 sequences from other cases diagnosed in the same time period and context (323 from Madrid and the remaining 639 from elsewhere in Spain), we observed how they distributed along the tree in independent branches (Fig. 2, right). These data suggest that they corresponded to independent importations from the community to the ward.

Two of the strains were found each in a single patient (strains 4 and 5), ruling out the implication of these cases in the outbreak. The remaining three strains were involved in limited independent transmissions with 3, 3, and 5 cases in each cluster (clusters 1, 2, and 3, respectively; 0 to 1 single nucleotide polymorphism [SNPs] within each cluster; Fig. 2, left). The HCW (case E), initially thought to have been infected after a household exposure, was associated with cluster 1.

Strain distribution among the three rooms affected by the outbreak (rooms 1, 2, and 3) is shown in Fig. 1B. A one-strain-one-room association was not observed but, rather, a much more heterogeneous situation. Patients infected with different strains had sequentially stayed in the rooms (two, three, and three different strains identified for patients in rooms 1, 2, and 3, respectively). In addition, there were times at which patients with different strains shared the same room (Fig. 1B). From these data, patient-to-patient transmission within the same room or exposure to contaminated surfaces did not seem to be the only explanation for the nosocomial transmissions.

## DISCUSSION

Surveillance of SARS-CoV-2 transmission is particularly relevant in hospital environments where exposed subjects are more vulnerable (7). COVID-19 death rates associated with nosocomial infections have been reported to be high, ranging between 33% (3, 8) and 38% (https://www.krisp.org.za/news.php?id=421). Additionally, nosocomial transmission increases the risk of exposure to HCWs (3–5), who may become transmission vectors (4).

Some large nosocomial outbreaks occurred during the first wave of COVID-19, mainly attributed to a shortage of PPE and lack of clear guidance on prevention and control measures. Efforts have been made to increase our knowledge on the dynamics of SARS-CoV-2 nosocomial transmissions in the first wave (3–5, 9; https://www.krisp.org.za/news.php?id=421).

WGS has been crucial to clarify that the nature of certain outbreaks may differ from the initial assumptions. A nosocomial outbreak in Ireland, where several simultaneous independent outbreaks were at first suspected to involve up to nine different wards (3), was later confirmed to be a limited number of transversal outbreaks. The use of WGS at the beginning of the first COVID-19 wave left uncertainties regarding the outbreaks, even after genomic analysis (5; https://www.krisp.org.za/news.php?id=421). The current use of real-time genomic epidemiology has fully proved its benefits (6), probably due to the higher diversity acquired by the currently circulating SARS-CoV-2 strains in comparison to those in the first wave and a high potential to rule out relationships.

In the second COVID-19 wave, with secured access to PPE and hospital control measures implemented, understanding SARS-CoV-2 nosocomial transmission remains important. In our study, WGS sheds light on the true complexity of a COVID-19 nosocomial outbreak. Once WGS findings were included, the initial assumption of a single outbreak caused by a likely virulent strain (six patients developed pneumonia, and 27% had a fatal outcome), interpreted as caused by patient-to-patient transmission and a potential role of contaminated surfaces, provided a completely different perspective.

WGS revealed that five different strains were coinciding in the ward during the outbreak, three of them shared by more than one patient. This suggested independent simultaneous importations of several strains from the community, which were then subsequently nonsocomially transmitted. This hypothesis was supported by performing an integrated analysis of the sequences from the cases in the ward with representative sequences of other circulating strains from the community for the same time period.

Therefore, our analysis supported that five different strains had been introduced in the ward. When the outbreak occurred, the gastroenterology ward received COVID-19-free patients from the pulmonology and internal medicine departments. Our WGS-based findings indicate that the currently applied standard measures, only addressed to reduce transmission once the patients are in the ward, are not enough if they are not accompanied by additional controls to prevent the introduction of undiagnosed presymptomatic or asymptomatic COVID-19 cases.

The five introduced strains coincided in time and involved patients staying in three neighboring rooms. Despite this spatial-temporal coincidence, only half of the strains were further transmitted, and the rest did not cause any secondary cases in the ward. These findings suggest the likely existence of singular, specific factors responsible for the outbreak rather than a general major systematic control deficiency.

The restriction of the outbreak to three neighboring rooms, with the sequential turnover of different strains in the cases who had occupied the rooms, minimizes the initially assumed possibility of contaminated surfaces. Although the primary transmission mode of SARS-CoV-2 in hospitals is close contact and exposure to droplets or contaminated fomites, our findings suggest airborne transmission. Air turnover was checked in the ward, and room 1 showed the lowest values of inward airflow. Room 3 was not connected to the general ventilation system to impulse air into the room; instead, a fan coil had been installed that could be manually switched off (following inspection, it was disconnected). Finally, room 2 was adjacent to the one with the lowest pressure of inward airflow (room 1), which may cause airflow distortions when doors are kept open. These data suggest that possible deficiencies in the air system may be a contributing factor for the concentration of cases in the three indicated rooms.

This study shows the importance of WGS-based analysis to correctly understand the true complexity behind nosocomial transmission events. This technique provided key data to interpret the here-reported outbreak, and the involvement in the outbreak of an HCW, initially thought to have acquired the infection outside the hospital, was only understood once WGS data were available.

We report a complex epidemiological scenario of a nosocomial COVID-19 outbreak in the second wave based on WGS. Initially, standard epidemiological findings led us to assume a homogeneous outbreak caused by a single SARS-CoV-2 strain, suspected to be somehow virulent due to the patients’ outcomes, driven by person-to-person transmission and contaminated surfaces. The discriminatory power of WGS offered a strikingly different perspective consisting of five importations of different strains, with only half of them causing secondary cases in three independent overlapping clusters and with a turnover of strains within the same rooms that ruled out the role of contaminated sources.

## MATERIALS AND METHODS

### Clinical data.

This was a retrospective study in a tertiary referral hospital in Madrid (Spain) that included all consecutive patients diagnosed with COVID-19 at admission (15 September to 12 October 2020) in a non-COVID-19 gastroenterology ward, as well as the health care workers (HCWs) who were in charge of these patients and diagnosed during the study period. Baseline characteristics and clinical and laboratory parameters of the patients at COVID-19 diagnosis and their outcome were obtained from their electronic medical records. Data were analyzed with the SPSS 20.0 package (SPSS Inc., Chicago, IL, USA). Numeric variables are expressed as medians and interquartile ranges (IQRs) and categorical variables as the number of cases and their percentages.

### Diagnostic RT-PCRs.

Viral RNA was extracted and purified from 300 μl of nasopharyngeal exudates with the aid of the KingFisher (Thermo Fisher Scientific, Waltham, MA) instrument. Next, an RT-PCR was performed, using the TaqPath COVID-19 CE-marked for *in vitro* diagnosis (CE-IVD) RT-PCR kit (Thermo Fisher Scientific, USA).

### Whole-genome sequencing.

Eleven microliters of RNA were used as the template for reverse transcription using Invitrogen SuperScript IV reverse transcriptase (Thermo Fisher Scientific, MA, USA) and random hexamers (Thermo Fisher Scientific, MA, USA). Whole-genome amplification of the coronavirus was done with an Artic_nCov-2019_V3 panel of primers (Integrated DNA Technologies, Inc., Coralville, IA, USA) (https://artic.network/ncov-2019) and the Q5 Hot Start DNA polymerase (New England Biolabs, Ipswich, MA, USA). Libraries were prepared using the Nextera Flex DNA library preparation kit (Illumina, Inc., CA, USA) following the manufacturer´s instructions.

Libraries were quantified with the Quantus fluorometer (Promega, WI, USA) before being pooled at equimolar concentrations (4 nM). Next, they were sequenced in pools of up to 17 libraries on the MiSeq system (Illumina, Inc., CA, USA) and the MiSeq Reagent Micro kit v2 (2× 151 bp) or in pools of up to 96 libraries with the MiSeq reagent (2× 201 bp).

FASTQ files above the Global Initiative on Sharing All Influenza Data (GISAID) thresholds were deposited at GISAID (EPI_ISL_654287, EPI_ISL_654285, EPI_ISL_654348, EPI_ISL_654204, EPI_ISL_654345, EPI_ISL_654357, EPI_ISL_654203, EPI_ISL_654176, EPI_ISL_654284, EPI_ISL_654292, EPI_ISL_654286, EPI_ISL_654294, EPI_ISL_654288, EPI_ISL_654351, and EPI_ISL_654349).

An in-house analysis pipeline was applied to analyze the sequencing reads. The pipeline can be accessed at https://github.com/pedroscampoy/covid_multianalysis. Briefly, the pipeline goes through the following steps: (i) removal of human reads with Kraken (10), (ii) preprocessing and quality assessment of FASTQ files using fastp v0.20.1 (11) (arguments, --cut tail, --cut-window-size, --cut-mean-quality, -max_len1, -max_len2) and FastQC v0.11.9 (http://www.bioinformatics.babraham.ac.uk/projects/fastqc), (iii) mapping with bwa v0.7.17 (12) and variant calling using IVAR v1.2.3 (13) using the Wuhan-1 sequence (GenBank accession no. NC_045512.2) as reference, and (iv) recalibration of punctual low-coverage positions using joint variant calling. When necessary, informative noncovered positions were analyzed by standard Sanger sequencing with the corresponding flanking primers from the Artic set.

### Ethics approval.

The study was approved by the ethical research committee of Gregorio Marañón Hospital (reference MICRO.HGUGM.2020-042). Informed consent was obtained, and consent for publication was obtained.
